# Supplementary material for: Predicting visual function by interpreting a neuronal wiring diagram
Source: Nature. 2024 Oct 2;634(8032):113–23. doi: 10.1038/s41586-024-07953-5 (PMC11446822; doi:10.1038/s41586-024-07953-5)
Supplement: Supplementary file 6 — CRF and ERF predictions for individual TmY4 and TmY9 cells. Analogous to Supplementary Data 3, but for TmY target types. Shown are the top four monosynaptic pathways, the strongest pathway passing through each of the top ten intermediary types (ranking from Extended Data Fig. 7), and the trisynaptic pathway Tm1–TmY–Dm3–TmY (see the section entitled Prediction of spatial normalization). [file 41586_2024_7953_MOESM6_ESM.zip › DataS4/TmY9q/720575940605810464.pdf]

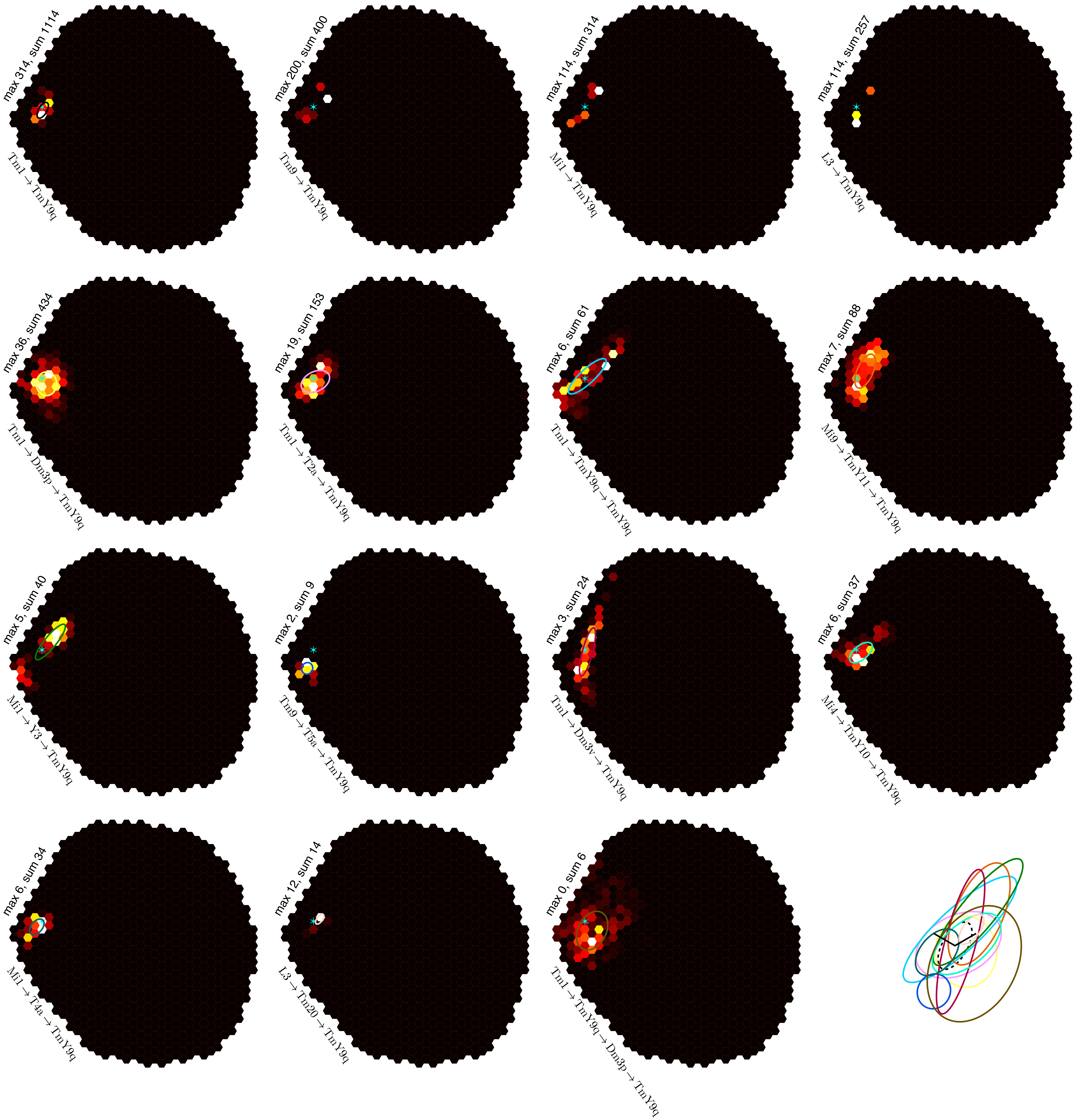
max 314, sum 1114
Tm1 → TmY9q
max 200, sum 400
Tm9 → TmY9q
max 114, sum 314
Mi1 → TmY9q
max 114, sum 257
L3 → TmY9q
max 36, sum 434
Tm1 → Dm3p → TmY9q
max 19, sum 153
Tm1 → T2a → TmY9q
max 6, sum 61
Tm1 → TmY9q → TmY9q
max 7, sum 88
Mi9 → TmY11 → TmY9q
max 5, sum 40
Mi1 → Y3 → TmY9q
max 2, sum 9
Tm9 → T5a → TmY9q
max 3, sum 24
Tm1 → Dm3v → TmY9q
max 6, sum 37
Mi4 → TmY10 → TmY9q
max 6, sum 34
Mi1 → T4a → TmY9q
max 12, sum 14
L3 → Tm20 → TmY9q
max 0, sum 6
Tm1 → TmY9q → Dm3p → TmY9q
